# Supplementary material for: Adverse Effect of the Duration of Antibiotic Use Prior to Immune Checkpoint Inhibitors on the Overall Survival of Patients with Recurrent Gynecologic Malignancies
Source: Cancers (Basel). 2023 Dec 7;15(24):5745. doi: 10.3390/cancers15245745 (PMC10742258; doi:10.3390/cancers15245745)
Supplement: Supplementary file 1 [file cancers-15-05745-s001.zip › cancers-2657273-supplementary.pdf]

Supplementary Materials:

Supplementary Table S1. Initial stage according to the primary tumor site.

Tumor sites	Initial stage				Total
	**I**	**II**	III	IV	
Endometrium	11 (30.6%)	6 (16.7%)	10 (27.8%)	9 (25.0%)	36 (100%)
Ovary	3 (2.7%)	9 (8.0%)	70 (62.5%)	30 (26.8%)	112 (100%)
Cervix	15 (28.3%)	17 (32.1%)	13 (24.5%)	8 (15.1%)	53 (100%)
Others	9 (64.3%)	2 (14.3%)	3 (21.4%)	0	14 (100%)

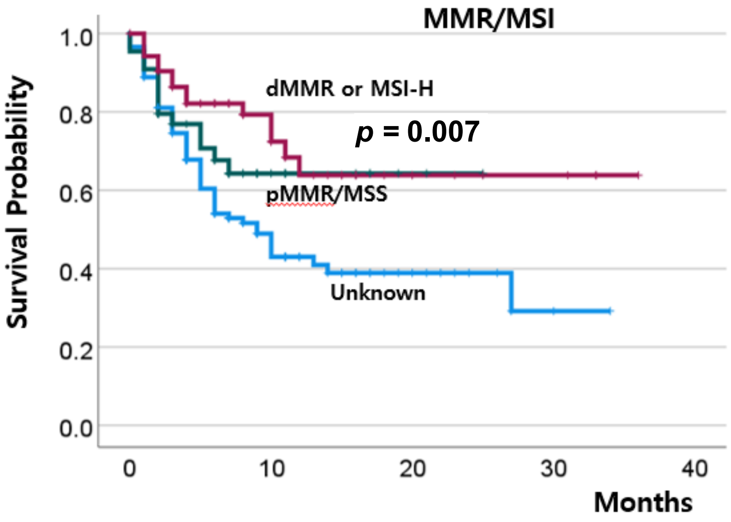

Supplementary Figure S2. Overall survival according to the MMR/MSI status dMMR, deficient mismatch repair; pMMR, proficient mismatch repair; MSI-H, microsatellite instability high; MSS, microsatellite instability stable.

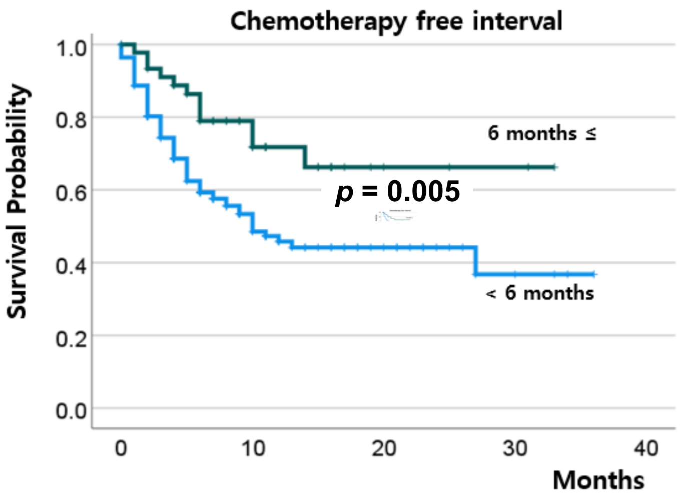

Supplementary Figure S3. Overall survival according to the chemotherapy-free interval.

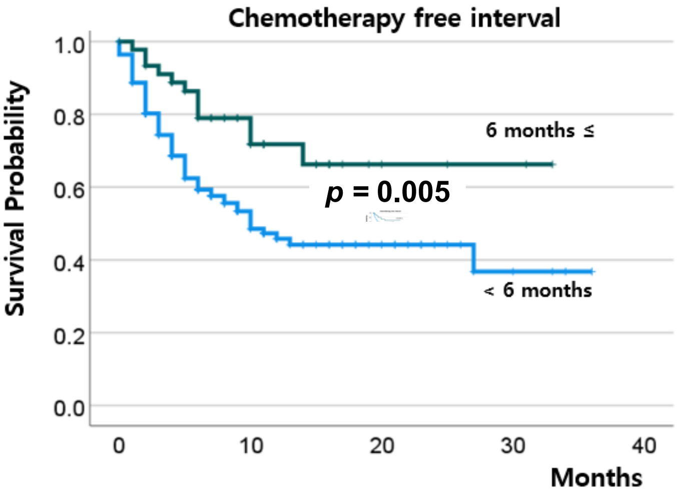

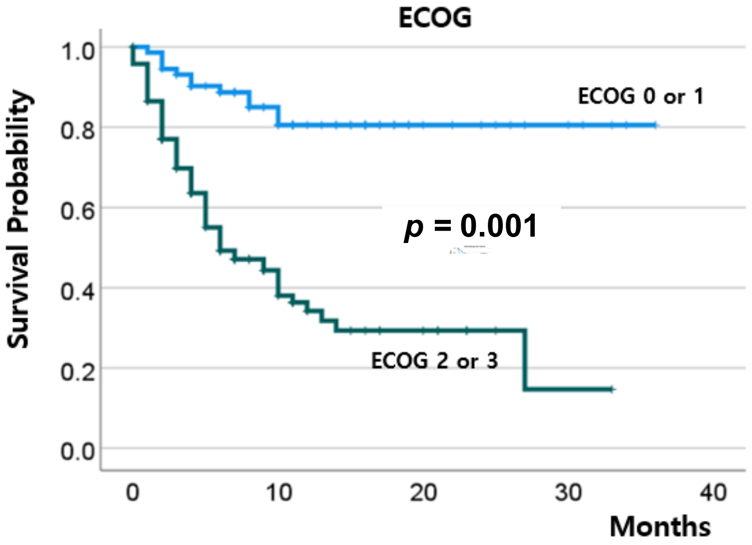

Supplementary Figure S4. Overall survival according to the ECOG status.

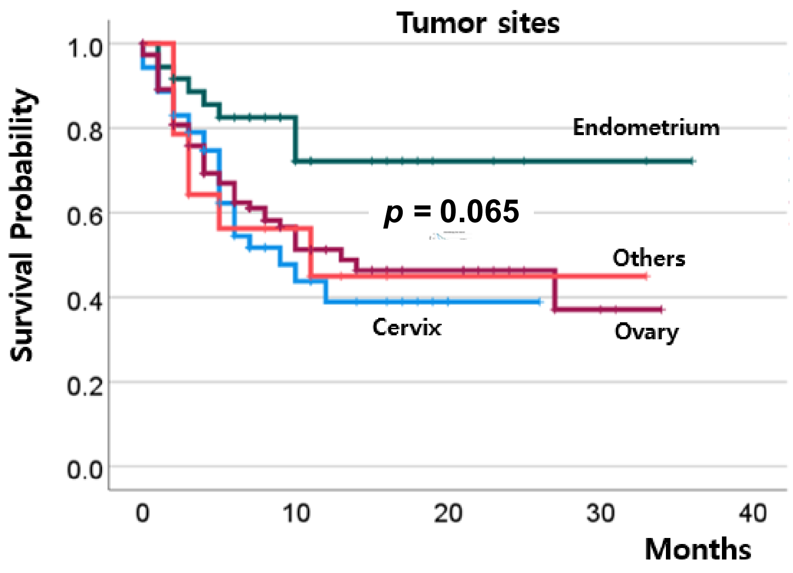

Supplementary Figure S5. Overall survival according to the tumor site (the others include the vulva and vagina).

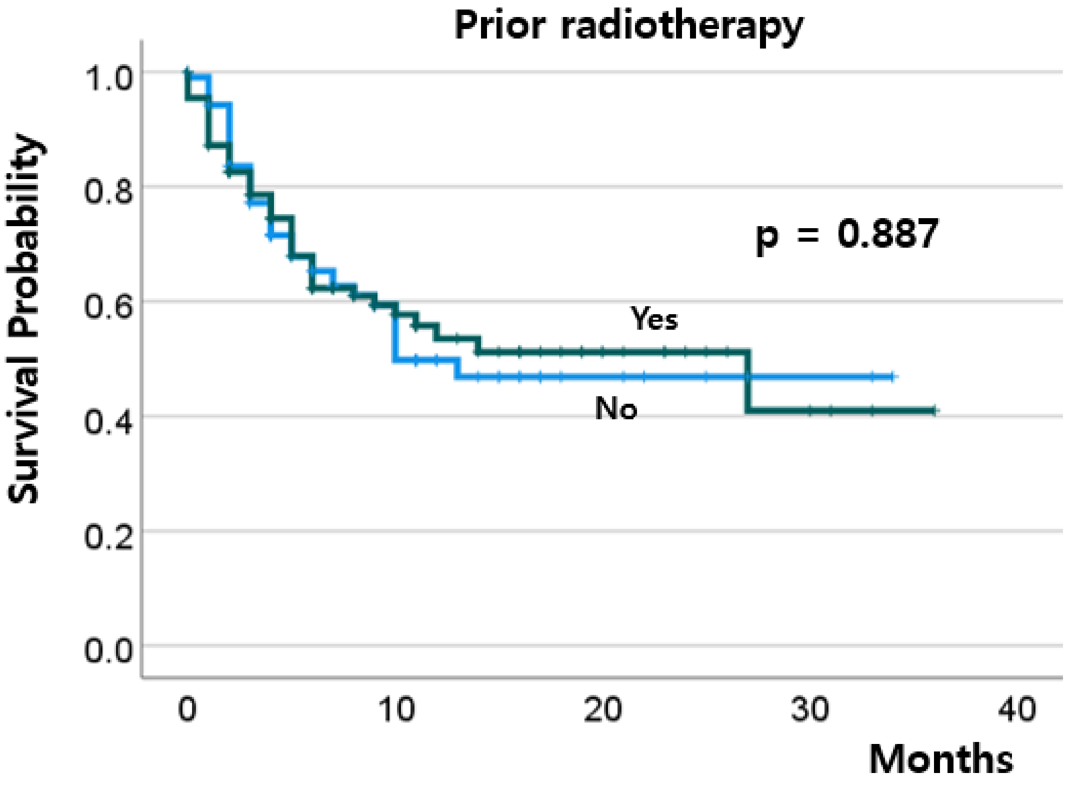

Supplementary Figure S6. Overall survival according to prior radiotherapy.

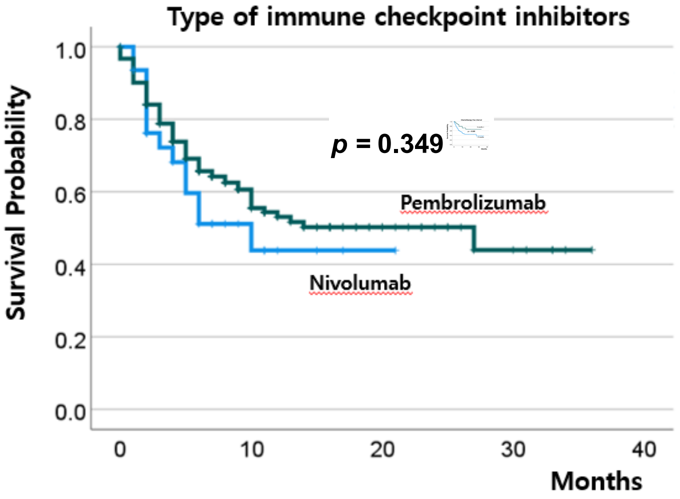

Supplementary Figure S7. Overall survival according to the type of immune checkpoint inhibitor.

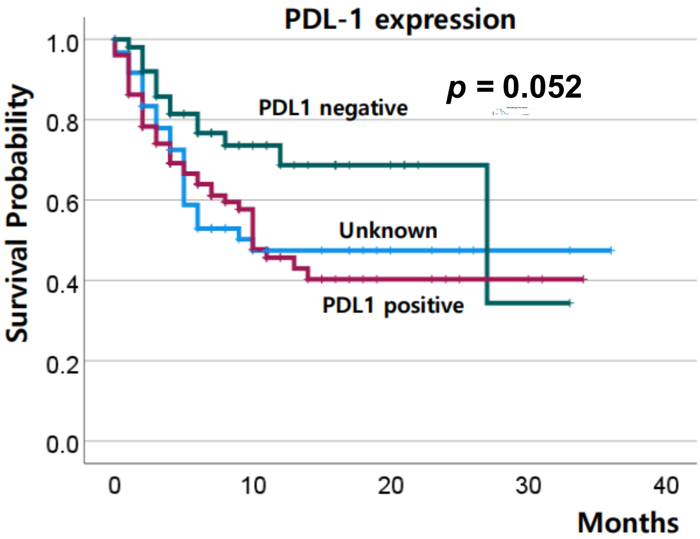

Supplementary Figure S8. Overall survival according to the PD-L1 expression.

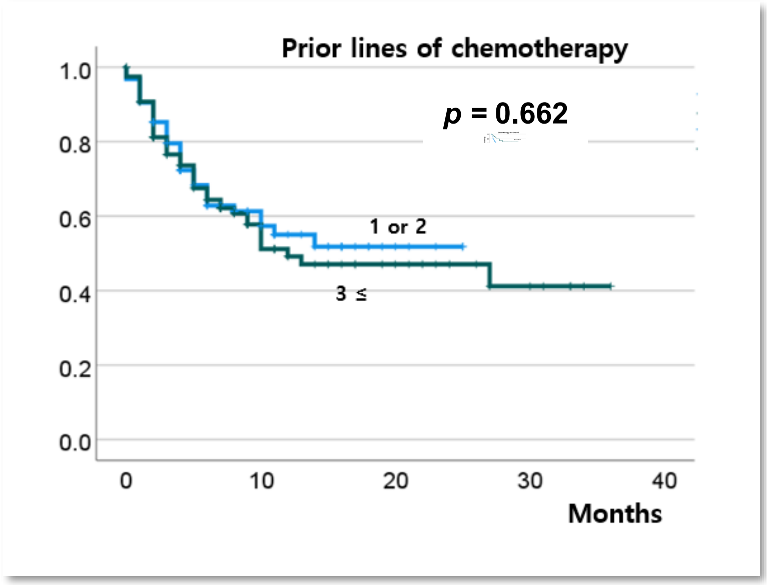

Supplementary Figure S9. Overall survival according to the prior lines of chemotherapy.

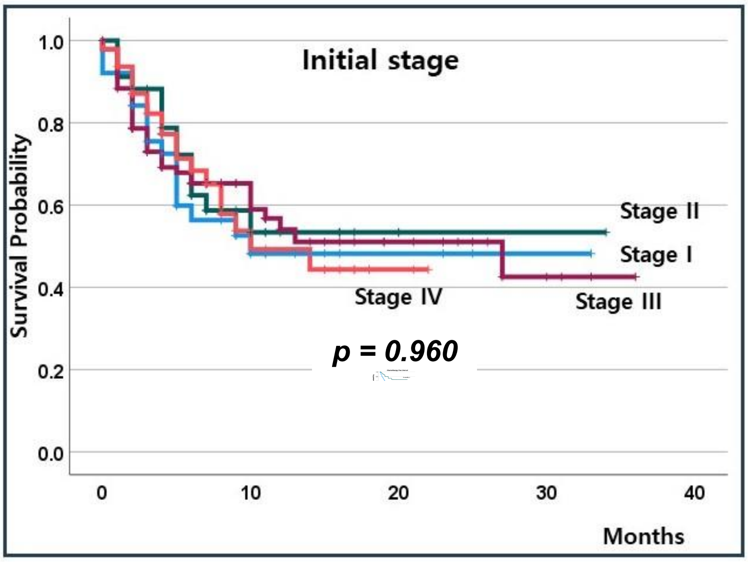

Supplementary Figure S10. Overall survival according to the initial stage.

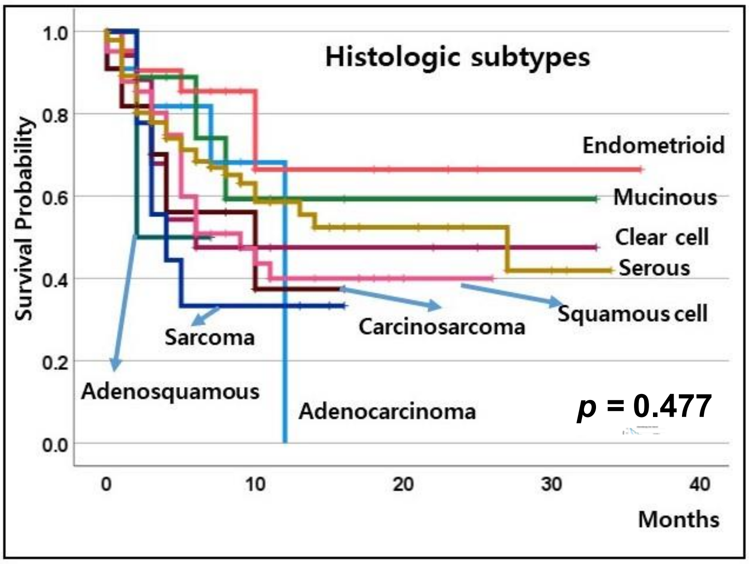

Supplementary Figure S11. Overall survival according to the histologic type.
